# Calibrating E-values for MS^2 ^database search methods

**DOI:** 10.1186/1745-6150-2-26

**Published:** 2007-11-05

**Authors:** Gelio Alves, Aleksey Y Ogurtsov, Wells W Wu, Guanghui Wang, Rong-Fong Shen, Yi-Kuo Yu

**Affiliations:** 1 National Center for Biotechnology Information, National Library of Medicine, NIH, Bethesda, MD 20894, USA; 2 Proteomics Core Facility, National Heart, Lung, and Blood Institute, NIH, Bethesda, MD 20892, USA

## Abstract

**Background:**

The key to mass-spectrometry-based proteomics is peptide identification, which relies on software analysis of tandem mass spectra. Although each search engine has its strength, combining the strengths of various search engines is not yet realizable largely due to the lack of a unified statistical framework that is applicable to any method.

**Results:**

We have developed a universal scheme for statistical calibration of peptide identifications. The protocol can be used for both *de novo *approaches as well as database search methods. We demonstrate the protocol using only the database search methods. Among seven methods -SEQUEST (v27 rev12), ProbID (v1.0), InsPecT (v20060505), Mascot (v2.1), X!Tandem (v1.0), OMSSA (v2.0) and RAId_DbS – calibrated, except for X!Tandem and RAId_DbS most methods require a rescaling according to the database size searched. We demonstrate that our calibration protocol indeed produces unified statistics both in terms of average number of false positives and in terms of the probability for a peptide hit to be a true positive. Although both the protocols for calibration and the statistics thus calibrated are universal, the calibration formulas obtained from one laboratory with data collected using either centroid or profile format may not be directly usable by the other laboratories. Thus each laboratory is encouraged to calibrate the search methods it intends to use. We also address the importance of using spectrum-specific statistics and possible improvement on the current calibration protocol. The spectra used for statistical (*E*-value) calibration are freely available upon request.

**Open peer review:**

Reviewed by Dongxiao Zhu (nominated by Arcady Mushegian), Alexey Nesvizhskii (nominated by King Jordan) and Vineet Bafna. For the full reviews, please go to the Reviewers' comments section.

## Introduction

Mass spectrometry (MS) based protein identification plays a key role in proteomics, with tandem MS (MS^2^) based peptide identification as an indispensable component. Knowing the importance of accurate identification of peptides from MS^2 ^spectra, numerous groups have devised what each group deems the best method for automated (computer-assisted) peptide identification. These methods include *de novo *types and database search types. Here we will focus on the database search type. Because each search method uses a different algorithm and proceeds from a different view of what spectrum components contain the most critical information for identification, the search results for one spectrum from various search engines may differ substantially. Furthermore, the statistical characterizations of various methods do not share a common foundation.

For example, methods such as Mascot [1], Sonar [2], InsPecT [3], OMSSA [4], and X!Tandem [5] provide directly an *E*-value or *P*-value for every hit reported. However, it is found that the *E*-values reported in these methods are not compatible: heuristic transformation is needed to transform the *E*-value reported by one method to the *E*-value reported by another [4]. Other methods – using correlation, posterior probabilities, score, or Z-score – include, but are not limited to, SEQUEST [6], MS-Tag [7], Scope [8], CIDentify [9], Popitam [10], ProbID [11], and PepSearch [12]. It is even harder to translate the statistical significance from any of these methods into other methods.

Although evaluations of various search engines have been documented in terms of the number of true positives found at a fixed false positive rate or in terms of total number of true positive hits found [13], these data, while important, do not provide us with the best cutoff to use for each search engine. Furthermore, if we fix the cutoff for a certain search engine, the evaluation information cannot tell us the equivalent cutoffs for other engines. Realizing the importance of reducing reported false identifications, journals such as Molecular & Cellular Proteomics have assembled guidelines attempting to establish a community standard in reporting protein identification [14-16]. The task of establishing a community standard, however, can best be achieved by having a common statistical framework.

This need for method-independent statistical characterization was well recognized by [17]. Assuming the high scoring tail of the score distribution can be approximated by pdf(S ≫ 1) ≈ C
 MathType@MTEF@5@5@+=feaafiart1ev1aaatCvAUfKttLearuWrP9MDH5MBPbIqV92AaeXatLxBI9gBaebbnrfifHhDYfgasaacPC6xNi=xH8viVGI8Gi=hEeeu0xXdbba9frFj0xb9qqpG0dXdb9aspeI8k8fiI+fsY=rqGqVepae9pg0db9vqaiVgFr0xfr=xfr=xc9adbaqaaeGacaGaaiaabeqaaeqabiWaaaGcbaWenfgDOvwBHrxAJfwnHbqeg0uy0HwzTfgDPnwy1aaceaGae8NaXpeaaa@374B@ exp(-*λ*S), [17] proposed to extract the search-method-dependent parameters C
 MathType@MTEF@5@5@+=feaafiart1ev1aaatCvAUfKttLearuWrP9MDH5MBPbIqV92AaeXatLxBI9gBaebbnrfifHhDYfgasaacPC6xNi=xH8viVGI8Gi=hEeeu0xXdbba9frFj0xb9qqpG0dXdb9aspeI8k8fiI+fsY=rqGqVepae9pg0db9vqaiVgFr0xfr=xfr=xc9adbaqaaeGacaGaaiaabeqaaeqabiWaaaGcbaWenfgDOvwBHrxAJfwnHbqeg0uy0HwzTfgDPnwy1aaceaGae8NaXpeaaa@374B@ and *λ *by fitting the high scoring tail of a given score histogram with the proposed exponential distribution. Once the parameters are determined for a given spectrum analyzed by a certain method, one can obtain the *P*-value of a given score by integration. This procedure presumably can provide method-independent score statistics in terms of *P*-values. However, not every scoring scheme will result in a score distribution with an exponential tail. A simple monotonic score transform, say S → S
 MathType@MTEF@5@5@+=feaafiart1ev1aaatCvAUfKttLearuWrP9MDH5MBPbIqV92AaeXatLxBI9gBaebbnrfifHhDYfgasaacPC6xNi=xH8viVGI8Gi=hEeeu0xXdbba9frFj0xb9qqpG0dXdb9aspeI8k8fiI+fsY=rqGqVepae9pg0db9vqaiVgFr0xfr=xfr=xc9adbaqaaeGacaGaaiaabeqaaeqabiWaaaGcbaWaaOaaaeaacqqGtbWuaSqabaaaaa@2D1B@, will bring an exponential tail immediately to a Gaussian tail. Furthermore, there is no guarantee that this method will provide *realistic *statistics for all methods. To be specific, it is not yet established that for a given *E*-value cutoff *E*_*c *_the average number of cumulative false positives ⟨*FP *(*E *≤ *E*_*c*_)⟩ will satisfy ⟨*FP*⟩ = *E*_*c*_, as anticipated from the definition of *E*-value.

Nevertheless, we do agree with the fundamental idea of [17]: the best way to build a common statistical standard is to start with a statistical quantity whose precise definition is independent of the details of the various search algorithms. Both the *P*-value and the *E*-value are appropriate for this purpose (see next section for more detail). For database searches, we use the *E*-value because it reflects directly the number of false positives expected. Although different search methods use different scoring schemes and have different quality scores, the search results can be compared once they are transformed into *E*-values. Unfortunately, it appears that the *E*-values reported by different search methods are not compatible. Furthermore, there are plenty of search engines that do not report *E*-values. The lack of a common statistical standard motivates us to provide a standard protocol to calibrate the *E*-values of various search engines. Although we only examine seven search engines in this paper, the protocol can be applied to any database search method. It is important to stress that different laboratories may adjust their instruments differently and perform the experiments differently. Thus each laboratory should separately calibrate the search methods they intend to use. However, once the calibration is done for each laboratory, it becomes sensible to compare the search results obtained at different laboratories.

The goal of this paper is to provide a universal protocol for statistical calibration. Although the pre-calibrated statistics of various methods are shown, these should not be viewed or used as a performance comparison. In the remainder of this paper, we will discuss the issue of a common statistical standard together with methods to implement it. After analyzing the intermediate results, we will then present the full results followed by discussion and summary.

## Method

Both the *E*- and *P*-values may be viewed as monotonically decreasing functions of some algorithm-dependent quality score S. For a given quality score cutoff, the *E*-value is defined as the expected number of hits in a random database with quality score greater than or equal to the cutoff. Similarly, *P*-value refers to the probability of finding a random hit with quality score greater than or equal to the cutoff.

To demonstrate our protocol, we have chosen to acquire spectral data in the profile (raw-data) format. We choose to use profile spectra because we believe such spectra contain more information. Further, this type of data is also preferred by a number of *de novo *peptide identification methods such as [18,19]. One should also note that calibration using different data types follows exactly the same protocol, except that the calibration formulas that give rise to calibrated *E*-values may appear different.

### Material

A known mixture of 7 proteins (purchased from SIGMA) containing equimolar levels of *α*-lactalbumin (LALBA_BOVIN, P00711), lysozyme (LYSC_CHICK, P00698), *β*-lactoglobulin B (LACB_BOVIN, P02754), hemoglobin (HBA_HUMAN and HBB_HUMAN, P69905 and P68871), bovine serum albumin (ALBU_BOVIN, P02769), apotransferrin (TRFE_HUMAN, P02787), and *β*-galactosidase (BGAL_ECOLI, P00722) was used for all experiments. Note that both the *α *chain and the *β *chain of hemoglobin were included. The protein mixture in 50 mM ammonium bicarbonate buffer was reduced with 10 mM DTT at 60°C for 1 hr, alkylated with 55 mM iodoacetamide at room temperature in the dark for 30 min, and digested with trypsin (Promega) at 50:1 mass ratio at 37°C overnight, as described in [20]. Three different levels of protein mixture -50 femtomoles, 500 femtomoles, and 5 picomoles of each protein – were then injected into LC/MS/MS resulting to effective concentrations of 10 nM, 100 nM and 1000 nM respectively. Two different kinds of mass spectrometers were utilized in this study: nanospray (NSI)/LTQ FT (Thermo Finnigan) and matrix assisted laser desorption ionization (MALDI)/TOF/TOF (Applied Biosystems). All data were acquired in the profile (raw-data) format.

For NSI/LTQ FT, following the procedure of [21], peptides were first loaded onto a trap cartridge (Agilent) at a flow rate of 2 *μ*l/min. Trapped peptides were then eluted onto a reversed-phase PicoFrit column (New Objective) using a linear gradient of acetonitrile (0–60%) containing 0.1% FA. The duration of the gradient was 20 min at a flow rate of 0.25 *μ*l/min, which was followed by 80% acetonitrile washing for 5 minutes. The eluted peptides from the PicoFrit column were nano-sprayed into an LTQ FT mass spectrometer. The data-dependent acquisition mode was enabled, and each survey MS scan was followed by five MS/MS scans with dynamic exclusion option on. The spray voltage and ion transfer tube temperature were set at 1.8 kV and 160°C, respectively. The normalized collision energy was set at 35%. Three different combinations of mass analyzers (LTQ LTQ, LTQ FT, and FT FT) were used to acquire protein mixtures at each level. For MALDI/TOF/TOF, following the procedure of [22], peptide separation was performed on a Famos/Switchos/Ultimate chromatography system (Dionex/LC Packings) equipped with a Probot (MALDI-plate spotting device). Peptides were injected and captured onto a trap column (PepMap C18, 5 *μ*m, 100 A, 300 *μ*m i.d. *× *5 mm) at 10 *μ*l/min. Peptide separation as achieved on an analytical nano-column (PepMap C18, 3 *μ*m, 100 A, 75 *μ*m i.d. *× *15 cm) using a gradient of 5 to 60% solvent B in A over 90 min (solvent A: 100% water, 0.1% TFA; solvent B: 80% acetonitrile/20% water, 0.1% TFA), 60 to 95% solvent B in A for 1 min, and then 95% solvent B for 19 min at a flow rate of 0.16 *μ*l/min. The HPLC eluant was supplemented with 5 mg/ml *α*-cyano-4-hydroxycinnamic acid (in 50/50 acetonitrile/water containing 0.1% TFA) from a syringe pump at a flow rate of 1 *μ*l/min, and spotted directly onto the ABI 4700 576-well target plates using the Probot. MALDI/TOF/TOF data were acquired in batch mode. To have a good sample of various instrument types and various concentrations, the number of spectra from each concentration and instrument type was made as close as possible. However, the number of spectra obtained from FT/FT and TOF/TOF was significantly smaller than that obtained from LTQ/FT and LTQ/LTQ. Therefore, we ended up using all the FT/FT and TOF/TOF data obtained and in the end selected ten thousand spectra with breakdown given in Table 1. The seven search methods studied in this paper are: SEQUEST (v27 rev12), ProbID (v1.0), InsPecT (v20060505), Mascot (v2.1), X!Tandem (v1.0), OMSSA (v2.0) and the newly developed RAId_DbS [23,24]. The last four methods provide *E*-values; the quality scores for the first three are X-correlation, posterior probability and MQ_score respectively.

**Table 1:** Breakdown of spectra used for *E*-value calibration

	FT/FT	TOF/TOF	LTQ/FT	LTQ/LTQ
1000 nM	207	1623	840	1624
100 nM	240	351	966	1624
10 nM	169	211	522	1623

Breakdown of spectra used for *E*-value calibration. Each entry in the matrix represents the number of spectra from a certain concentration (row) and an instrument type (column).

### Random Database Construction

The random database is constructed using the following protocol. First, one generates a string A
 MathType@MTEF@5@5@+=feaafiart1ev1aaatCvAUfKttLearuWrP9MDH5MBPbIqV92AaeXatLxBI9gBaebbnrfifHhDYfgasaacPC6xNi=xH8viVGI8Gi=hEeeu0xXdbba9frFj0xb9qqpG0dXdb9aspeI8k8fiI+fsY=rqGqVepae9pg0db9vqaiVgFr0xfr=xfr=xc9adbaqaaeGacaGaaiaabeqaaeqabiWaaaGcbaWenfgDOvwBHrxAJfwnHbqeg0uy0HwzTfgDPnwy1aaceaGae8haXheaaa@3747@ of amino acids of specified length. The probability of occurrence for each amino acid is given by its corresponding Robinson-Robinson frequency [25], the collection of which is a well accepted standard for amino acid background frequencies. However, our random database generating routine can also take user-specified amino acid frequencies to generate the long string of amino acids. No correlation between residues is attempted.

After the long string of amino acids is generated, we remove *true positive *peptides, if any, from the string. As a concrete example, we assume that the digesting enzyme used is trypsin. There are thus two possible cleavage points: the C-terminals of lysine (K) and arginine (R). We will call both K and R *C-cleaving *residues. For every one of the eight protein chains from seven target proteins, we look for all the C-cleaving residues starting from the C-terminal end and label the *i*th residue found by *c*_*i*_. For any two consecutive C-cleaving residues, *c*_*i *_and *c*_*i*+1_, with separation greater than five residues, the peptide [*b*_*i*+1_,..., *c*_*i*_] (where *b*_*i*+1 _is the C-terminal neighbor of *c*_*i*+1_) is searched for in the random database and removed from the random database if present. Each removal will fragment one string into two. If the separation between two consecutive C-cleaving residues, *c*_*i *_and *c*_*i*+1_, is less than five amino acids, the peptide used for the purpose of removing true positive purpose is given by the five letter peptide starting from a residue that is five letter away from *c*_*i *_in the N-terminal direction. This peptide will include both *c*_*i*+1 _and *c*_*i*_. We ignore the proline rule, however, because this neglect does not create a danger of keeping a true positive peptide in the string A
 MathType@MTEF@5@5@+=feaafiart1ev1aaatCvAUfKttLearuWrP9MDH5MBPbIqV92AaeXatLxBI9gBaebbnrfifHhDYfgasaacPC6xNi=xH8viVGI8Gi=hEeeu0xXdbba9frFj0xb9qqpG0dXdb9aspeI8k8fiI+fsY=rqGqVepae9pg0db9vqaiVgFr0xfr=xfr=xc9adbaqaaeGacaGaaiaabeqaaeqabiWaaaGcbaWenfgDOvwBHrxAJfwnHbqeg0uy0HwzTfgDPnwy1aaceaGae8haXheaaa@3747@.

After this true positive removal procedure, the original random string A
 MathType@MTEF@5@5@+=feaafiart1ev1aaatCvAUfKttLearuWrP9MDH5MBPbIqV92AaeXatLxBI9gBaebbnrfifHhDYfgasaacPC6xNi=xH8viVGI8Gi=hEeeu0xXdbba9frFj0xb9qqpG0dXdb9aspeI8k8fiI+fsY=rqGqVepae9pg0db9vqaiVgFr0xfr=xfr=xc9adbaqaaeGacaGaaiaabeqaaeqabiWaaaGcbaWenfgDOvwBHrxAJfwnHbqeg0uy0HwzTfgDPnwy1aaceaGae8haXheaaa@3747@ has been fragmented into many shorter pieces, each of which is regarded as a random protein. Furthermore, it is guaranteed that any possible peptide resulting from digesting the seven standard proteins with trypsin is not present in the random database. Therefore, any hit from the random database is a *false positive*. Eight random databases -three of 1 giga residues (10^9 ^amino acids), three of 100 mega residues (1 mega residues ≡ 10^6 ^amino acids), and two of 10 mega residues – were constructed for future testing.

## Analysis and results

Once the random databases were constructed, each individual search engine analyzed the ten thousand spectra and searched for peptide hits in the random protein databases. Since the random databases contain no true positives, all hits found were false positives. Because each search engine reports either a quality score or an *E*-value for each hit found, we then obtained the average number of false positives with *E*-value smaller than (or quality score greater than) the given cutoff.

For search methods reporting *E*-values, it is well known that the smaller the *E*-value the better the identification confidence. For quality scores, the trend is the opposite: the higher the quality score, the better the identification confidence. For the sake of uniformity, we introduce a database size dependent effective variable *x*_*db*_, which is simply the *E*-value for search methods reporting *E*-values and is a monotonically decreasing function of quality score, *e*^-(Quality score)^, for search methods that do not return *E*-values; see Table 2.

**Table 2:** Scaling of the database size and functional transformation to yield calibrated *E*-values.

Method	reported	*x*_db_	*x*_1gr _= *x*_db _(1gr/db_size)^*α*^	*E *= *f*(*x*_1gr_)
SEQUEST	X-correlation	*x*_db _= *e*^-(X-correlation)^	*x*_1gr _= *x*_db _(1gr/db_size)^-0.176^	*f*(*x*_1gr_) = *e*^10.59 ^*x*_1gr_^4.11^
InsPecT	MQ_score	*x*_db _= *e*^-(MQ_score)^	*x*_1gr _= *x*_db _(1gr/db_size)^-1^	*f*(*x*_1gr_) = [*e*^16.08 ^*x*_1gr _^1.4^]^1/2 ^if *x*_1gr _≥ 0.148
				*f*(*x*_1gr_) = [*e*^2.78 ^*x*_1gr _^0.48^]^1/2 ^if *x*_1gr _> 0.148
ProbID	Posterior Prob.	*x*_db _= *e*^-(Posterior Prob.)^	*x*_1gr _= *x*_db _(1gr/db_size)^-5^	*f*(*x*_1gr_) = *e*^13.82 ^*x*_1gr _^0.17^
Mascot	*E*-value	*x*_db _= *E*-value	*x*_1gr _= *x*_db _(1gr/db_size)^0.301^	*f*(*x*_1gr_) = *x*_1gr_/225
X!Tandem	*E*-value	*x*_db _= *E*-value	*x*_1gr _= *x*_db_	*f*(*x*_1gr_) = *x*_1gr_/10
OMSSA	*E*-value	*x*_db _= *E*-value	*x*_1gr _= *x*_db _(1gr/db_size)^-0.301^	*f*(*x*_1gr_) = *e*^-2.26 ^*x*_1gr _^0.57^
RAId_Dbs	*E*-value	*x*_db _= *E*-value	*x*_1gr _= *x*_db_	*E *= *f*(*x*_1gr_) = *x*_1gr_

Scaling of the database size and functional transformation to yield calibrated *E*-values. The calibration is done in two steps: first transform a database-size-dependent variable *x*_db _(e.g. *e*^-(X-correlation) ^for SEQUEST and *E*-value for X!Tandem) into a standard one, *x*_1gr_, which is calibrated for database size of 1 giga residues; second, a functional transformation *f*(*x*_1gr_) to yield the correct *E*-value given the equivalent variable *x*_1gr_. The symbol db_size, as suggested by its name, represents the size of the database used in a search. Note that the size scaling and the functional transformations may vary from laboratory to laboratory. However, once calibrated, it becomes sensible to compare the search results from different laboratories using different machines and different analysis software.

For all search methods tested, except Mascot, we ran each spectrum against all eight random databases of three different sizes. It was found that in general there is little difference in the number of false positives found with a given cutoff when using random databases of the same size. We also observed an interesting trend: the variation within the same size decreases as the database size increases. Due to limitation on the local availability and speed, it was not feasible to run Mascot on all eight random databases. For Mascot, we ran the 10, 000 spectra against a single 1 giga-residue random database instead of all three of them. The databases of smaller sizes were all used. Since the set-to-set variation decreases with random database size, we believe having only one run using a 1 giga-residue random database does not greatly increase the uncertainty in the statistical accuracy of Mascot after calibration.

**Figure 1:**
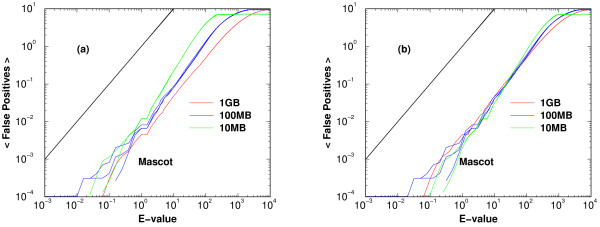
**An example of *E*-value calibration with database size dependence**. Average cumulative number of false positives versus *E*-values for Mascot. Theoretically speaking, if the number of trials is large enough, the average number of false positives with *E*-value less than or equal to a cutoff *E*_*c *_should be *E*_*c *_(indicated by the solid black line). Panel (a) displays the results from various random databases of different sizes; panel (b) displays the same results after rescaling the effective variable according to the size of the random databases. For Mascot, the rescaled curves aggregate well and the aggregate shows a slope quite parallel to the theoretical curve on the log-log plot, indicating a simple rescaling of *E*-value will suffice to bring the aggregate to the theoretical line. For other methods, the transformation function may be more complicated.

A larger difference may appear, however, amongst results obtained from searching in random databases of different sizes. Except for X!Tandem and RAId_DbS, all search methods require a size normalization in their effective variable *x*_db _in order to collapse the curves in the ⟨*FP*⟩ versus *x*_db _plot, where ⟨*FP*⟩ represents the average number of false positives with effective variable less than or equal to *x*_db_. As a consequence, the first step we took was to normalize the effective variable to a fixed size, 1 giga residues. Panel (a) of Fig. 1 displays the ⟨*FP*⟩ versus *E*-value for search results using random databases of different sizes. After a size normalization to 1 giga residues, *x*_1gr _= *x*_db _*× *(1gr/db_size)^0.301^, the data obtained from random databases of various sizes collapse well (panel (b)). Furthermore, the aggregated curves form a bundle with slope quite parallel to the theoretical curve on the log-log plot, indicating that one only needs a simple rescaling to bring the aggregate to the theoretical curve. It may not always be this easy for every search engine. However, the basic idea of performing the calibration is very straightforward: (1) the scale needed to transform the effective variable from a given database size to the size of 1 giga-residue is assumed to be of the form (1gr/db_size)^*α*^, or more precisely *x*_1gr _= *x*_db_(1gr/db_size)^*α *^with *α *determined by best fitting; (2) approximate the relationship between the logarithm of the scaled variable *x*_1gr _and the average number of false positives by linear segments.

The size normalizations and the transformation functions, giving rise to the correct *E*-value for given effective variables of various methods, are documented in Table 2. The scaling assumption made for effective variable is heuristic and is not guaranteed to hold true for all search methods yet to come. When this assumption breaks down, however, all one needs is to consider a more general function *g*(*x*_db_, 1gr/db_size) to relate *x*_1gr _and *x*_db_. Of course, the condition *g*(*x*, *y *= 1) = *x *has to be imposed then. As for the linear segment approximation between the logarithm of the scaled variable *x*_1gr _and ⟨*FP*⟩, it can be replaced by a tabulation approach. That is, based on the data obtained from searching the random one may construct a table converting from the variable *x*_1gr _to the standardized *E*-value, *i.e. *⟨*FP*⟩.

**Figure 2:**
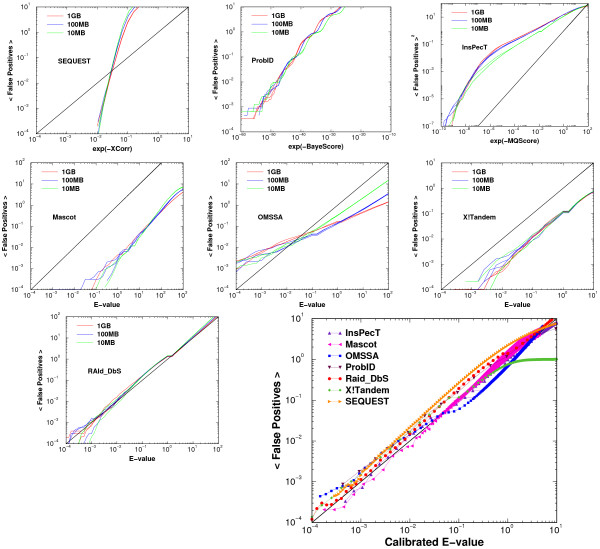
**Average cumulative number of false positives versus the effective variable to 1 giga residues**. The effective variable is *E*-value for search methods that report *E*-values, and becomes *e*^-(Quality score) ^for others. Except for X!Tandem and RAId_DbS, the effective variables of other methods do have a database size dependence and size calibrations are needed. Except for RAId_DbS, all other methods need calibration transformations to reach the *calibrated E*-value. Theoretically speaking, average number of false positives with *E*-values less than or equal to a cutoff *E*_*c *_should be *E*_*c *_provided that the number of trials is large enough. And this is how our *E*-value calibration is done. In the last panel, we plot the *calibrated E*-value as the abscissa and the average number of false positives with that *E*-value or smaller as the ordinate. The calibrations are able to bring the calibrated *E*-values close to the average number of false positives as theoretically expected.

In the first seven panels of Fig. 2, we show the aggregate of results obtained from up to eight random databases of three different sizes. Note that except for X!Tandem and RAID_DbS, all figures are obtained after one rescales the effective variable to its 1 giga-residue value. As shown in this figure, the aggregates for Mascot, X!Tandem, and RAId_DbS are reasonably parallel to the theoretical curve. In fact, not only quite parallel to the theoretical curve, RAId_DbS is also *very close *to it. As a consequence, RAId_DbS does not need additional transformation to retrieve correct *E*-values for peptide hits found. For SEQUEST, ProbID and OMSSA, the aggregate is fitted by a straight line on the log-log plot. For InsPecT, we plot along the ordinate ⟨*FP*⟩^2 ^instead of ⟨*FP*⟩. We approximate the aggregate by two straight lines depending on whether the effective variable is larger or smaller than a threshold 0.148. The [..]^1/2 ^expression in the last column of Table 2 reflects the fact that we need to take a square root before returning to ⟨*FP*⟩. The last panel of Fig. 2 deserves further elaboration. In this panel, each method is represented by a single curve. Each curve is obtained in two steps. After database size normalization, the average normalized false positives versus effective variable curves of a given method will become close. One first averages over those curves to obtain a single curve, based on which an approximate functional transformation is constructed to transform the effective variable to the calibrated *E*-value (see Table 2). One then goes through the results from the ten thousand spectra and transforms their quality scores or their original *E*-values into *calibrated E*-values. Plotting the calibrated *E*-value along the abscissa and average number of false positives as the ordinate yields a single curve in this panel. After our calibration, the statistical significance is unified and there is a universal standard.

As a specific example of how one may transform a method specific quality score to calibrated *E*-value, we demonstrate using SEQUEST. For a SEQUEST hit with X-correlation value 3.5 obtained by searching in a database of size 100 mega residues (implying db_size = 10^8 ^amino acids), this procedure is easily carried out using Table 2. First, from the third column we find that *x*_db _= *e*^-3.5 ^≈ 0.03. Then going to the fourth column we find *x*_1gr _= *x*_db_(10^9^/10^8^)^-0.176 ^≈ 0.03 × (2/3) ≈ 0.02. We then go to the last column and use

*E *= *f*(*x*_1gr_) = *e*^10.59^(*x*_1gr_)^4.11 ^≈ 0.00413.

That is, for a hit with X-correlation value 3.5, we end up obtaining a calibrated *E*-value of approximately 0.00413 if the search is done in a database of size 100 mega residues.

Finally, we also tested the accuracy of calibrated *E*-values in a different context, *i.e.*, from the perspective of true positives. Basically, the *E*-value of a significant peptide hit may be interpreted as the probability of that peptide being a false positive when *E *is small. Using a more careful argument (see Appendix for details), one finds that

**Figure 3:**
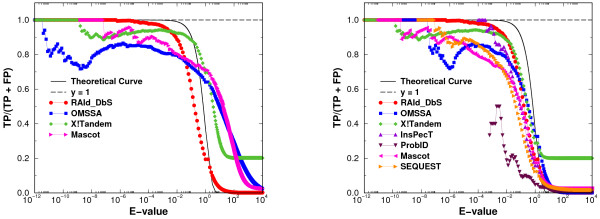
**Average ratio of true positives to the sum of true and false positives versus *E*-values**. In this plot, we show the assessment of the accuracy of calibrated *E*-values. The ratio *TP/*(*TP *+ *FP*) is plotted against *E*-value, see text for more details. The black solid curve corresponds to the theoretical line given by Eq. (1). In the left panel, we plot the results from four *E*-value-reporting search methods using the original *E*-values prior to calibrations. Not all the experimental curves are close to the theoretical curve. In the right panel, the *calibrated E*-values are used and one sees that the four curves from the left panel now form a much tighter bundle. Furthermore, even for methods that do not report *E*-value directly, except perhaps for ProbID we see they also fall within the bundle. The error bars in these plots are suppressed for clarity. They are about the size of the symbol at the low *E*-value end, and become larger (four to six times the symbol sizes) near large *E*-value end.

TP(E≤Ec)TP(E≤Ec)+FP(E≤Ec)=e−Ec,
 MathType@MTEF@5@5@+=feaafiart1ev1aaatCvAUfKttLearuWrP9MDH5MBPbIqV92AaeXatLxBI9gBaebbnrfifHhDYfgasaacPC6xNi=xI8qiVKYPFjYdHaVhbbf9v8qqaqFr0xc9vqFj0dXdbba91qpepeI8k8fiI+fsY=rqGqVepae9pg0db9vqaiVgFr0xfr=xfr=xc9adbaqaaeGacaGaaiaabeqaaeqabiWaaaGcbaqcfa4aaSaaaeaacqWGubavcqWGqbaucqGGOaakcqWGfbqrcqGHKjYOcqWGfbqrdaWgaaqaaiabdogaJbqabaGaeiykaKcabaGaemivaqLaemiuaaLaeiikaGIaemyrauKaeyizImQaemyrau0aaSbaaeaacqWGJbWyaeqaaiabcMcaPiabgUcaRiabdAeagjabdcfaqjabcIcaOiabdweafjabgsMiJkabdweafnaaBaaabaGaem4yamgabeaacqGGPaqkaaGaeyypa0Jaemyzau2aaWbaaeqabaGaeyOeI0Iaemyrau0aaSbaaeaacqWGJbWyaeqaaaaacqGGSaalaaa@5055@

where *TP*(*E *≤ *E*_*c*_) represents the number of true positives with *E*-value less than *E*_*c *_and *FP *(*E *≤ *E*_*c*_) represents the number of false positives with *E*-value less than *E*_*c*_. For each search engine, each of the ten thousand spectra was used to search for candidate peptides in the NCBI's nr database. Each candidate peptide was then classified as a true positive if it is a partial segment of the seven standard proteins used, and was classified as a false positive otherwise. We then binned the true positives and false positives according to the logarithm of the (calibrated) *E*-values. The ratio of the cumulative number of true positives to the sum of true and false positives is expected to follow the theoretical line (1) in Fig. 3.

In the left panel of Fig. 3, the results for the four search engines that report *E*-values are displayed. Note that, the abscissa is the original *E*-value reported by each search engine prior to calibration. Further, curves from different search engines do not agree with each other, and most importantly, many of them are not in agreement with the theoretical curve. In the right panel of Fig. 3, the abscissa is now the *calibrated E*-value. Except for ProbID, curves from different search engines aggregate well and are much closer to the theoretical line. This indicates that it is indeed possible to calibrate the *E*-values for various search engines and the calibration, once done, can approximate well through (1) the probability that a candidate peptide is a true positive.

**Figure 4:**
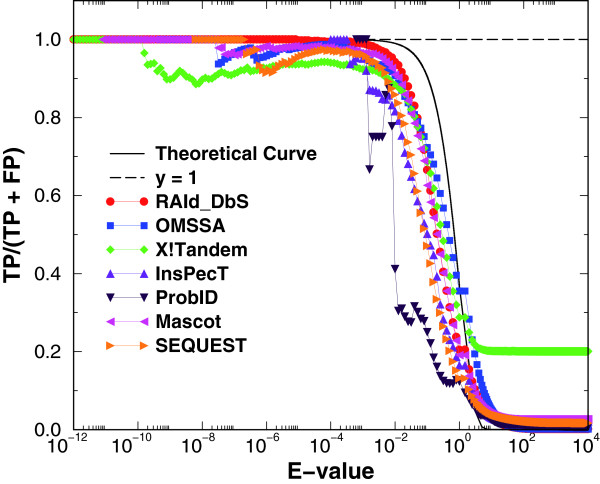
**Average ratio of true positives to the sum of true and false positives versus *E*-values with cluster removal employed**. This plot displays results obtained from using the NCBI's nr database but with cluster removal procedure (see text). The black solid curve corresponds to the theoretical line given by Eq. (1). As one may see, now almost all the experimental curves are close to the theoretical curve. In particular, even ProbID is now close to the theoretical curve when compared with the right panel of Fig. 3. Again, the error bars in these plots are suppressed for clarity. They are about the size of the symbol at the low *E*-value end, and become larger (four to six times the symbol sizes) near large *E*-value end.

A way to make a real protein database act as a random database is to perform a cluster removal procedure [23]. Basically, one uses the target proteins as the queries and removes from the database proteins with low *E*-values (say *E = *10^-15^) when aligning with any of the target proteins. This procedure is designed to eliminate *multiple-counting *of hits resulting from the fact that real proteins come in families of various sizes. The target proteins themselves, however, are excluded from removal. In Fig. 4 we display again the results of *TP/*(*TP *+ *FP*) with cluster removal invoked. When compared to the right panel of Fig. 3, the agreement between the theory and experimental data has improved. In particular, ProbID is now much closer to the rest of the search methods.

## Discussion and summary

We have shown in this paper that it is possible to build a unified statistical framework among the available peptide database search methods. The unified statistical significance, expressed in terms of *calibrated E*-values, is stable and independent of the search algorithm employed. Although we only apply the method to unify the statistics among seven peptide database search methods, the protocol provided is generic and can be applied to any other database search method to transform its quality score to a calibrated *E*-value. We recommend using 10, 000 spectra to calibrate each search method of interest. From our experience, this gives rise to *E*-value accuracy (after calibration) within three fold of the theoretical value throughout the range 10^-4 ^≤ *E *≤ 1. That is, *E*_theory_/3 ≤ *E*_calibrated _≤ 3*E*_theory_. Preferably, the 10, 000 spectra should be prepared from a mixture of various concentrations such as those described in the text.

There is room for improvement in our calibration protocol if the software developers are willing to provide more information other than reporting top hits. As we have been emphasizing, it is important to have *spectrum-specific *score statistics [19,23]. This is because the nature of noise depends on the peptide content as well as other numerous factors that eventually lead to spectrum-specific noise. When converting *scores *into *E*-values based on pooled results out of 10, 000 spectra searches, one looses spectrum specificity. Ideally, for each spectrum, one should infer from its score histogram to *E*-value assignment [23]. However, in addition to the score histogram, one will also need to *characterize *the score distribution. If this can be done, then one may bypass the need of calibration through random databases as described in this paper and enjoy the use of spectrum-specific statistics.

In our study, we found that most search methods, except for X!Tandem and RAId_DbS, report quality scores or *E*-values that are sensitive to the size of database searched. We have so far used database size of 1 giga residues as the base point for calibration. It is, however, straightforward to normalize the *effective *variable to other database size. This will then lead to a slight modification of Table 2 without changing, however, the final calibrated *E*-values.

Finally, we would like to emphasize that our statistical calibrations provide a unified framework to assign statistical significance for all available database search methods. Therefore, it is possible to take advantage of these unified statistics when one tries to combine search results from different search engines. This important task is beyond the scope of the current paper, but certainly deserves a serious investigation.

## Authors' contributions

GA designed the research, carried out the research, and analyzed the results. AO carried out the research and analyzed the results. WW, GW and RFS did the experiment and participated in data analysis. YKY designed the research, analyzed the results and wrote the paper.

## Reviewers' Comments

### Reviewer's Report 1

Sent to the reviewer on August 6th, 2007. Review report received on August 15th, 2007.

Review by Dongxiao Zhu (nominated by Arcady Mushegian) with contribution from Arcady Mushegian, Stowers Institute for Medical Research

1. It seems that calibrating P-values rather than E-values is more widespread in statistics and computational biology literature, the conventional way would be combining P-values using Chi-Square test, ie, (−2)∑i=1Kln⁡(Pi)~χ2(2K)
 MathType@MTEF@5@5@+=feaafiart1ev1aaatCvAUfKttLearuWrP9MDH5MBPbIqV92AaeXatLxBI9gBaebbnrfifHhDYfgasaacPC6xNi=xH8viVGI8Gi=hEeeu0xXdbba9frFj0xb9qqpG0dXdb9aspeI8k8fiI+fsY=rqGqVepae9pg0db9vqaiVgFr0xfr=xfr=xc9adbaqaaeGacaGaaiaabeqaaeqabiWaaaGcbaGaeiikaGIaeyOeI0IaeGOmaiJaeiykaKYaaabmaeaacyGGSbaBcqGGUbGBcqGGOaakcqWGqbaudaWgaaWcbaGaemyAaKgabeaakiabcMcaPaWcbaGaemyAaKMaeyypa0JaeGymaedabaGaem4saSeaniabggHiLdGccqGG+bGFiiGacqWFhpWydaahaaWcbeqaaiabikdaYaaakiabcIcaOiabikdaYiabdUealjabcMcaPaaa@4540@. For a more complete literature review, see to Larry Hedges and Ingram Olkin. Statistical Methods for Meta-Analysis. Journal of the American Statistical Association, Vol. 82, No. 397 (Mar., 1987), pp. 350–351. At pp. 2–3, authors mention one advantage of using P-values, but then abandoned the thought without explanation. Can authors provide the motivation for calibrating E-values rather than P-values?

2. The transformations proposed by the authors (see Table 2) are not well-explained in the text, and it is not even clear whether there has been any theory involved. For example, why the posterior probability generated in ProbID is calibrated using a simple exponential transformation – is it because of the statistical model employed in ProbID?

3. Page 7, 2nd paragraph from bottom, lines 2–3, "in general, there is little difference in the number of false positives found within a given cutoff when using random databases of the same size" – what are the actual numbers? And if this is so, why to calibrate the E-values in the first place?

4. Page 4, 4th line from top, P-value is not a probability but a test statistic.

5. The choice of Robinson and Robinson data for background frequencies seems a bit odd: is it not better to derive them from the database directly?

### Author's Response

1. Combining the *P*-values using the Fisher's formula is reasonable only if the items to be combined share the same statistical standard. The main purpose of this paper is to provide a protocol to reach a universal statistical standard. Only then can the combination of *P*-values become fruitful. In fact, we had already carried out the task of combining the statistical significance for various methods and are in the process of writing it up. As for the motivation to calibrate *E*-value instead of *P*-value, it is simply because for a given query spectrum, the effective database size used by various methods may be different. For example, some method may employ heuristics results in a smaller effective database size, while other methods may have different mass accuracy requirement that may also result in different effective database size. Since there is no way for users like us to know the effective database sizes used by various methods, we choose to calibrate the *E*-values.

2. The reviewer is correct that there is no real theory for converting the score of a search engine to *E*-values. This is largely due to the fact that most scoring methods employed in peptide identification are heuristic in nature and thus does not have *theoretically *characterizable statistics. The exponential transformation is chosen for two reasons. First, it gives a monotonic transformation. Second, heuristically it seems easier to compare with the average number of false positives.

3. The reviewer might have misunderstood the meaning of that sentence. The key point there is that the statistical behavior is robust across random databases of the same size. That is, for a given quality score cutoff, the numbers of false positives found within random databases of the same size are roughly the same. *This does not warrant anything about the correctness of the statistics*. For methods reporting *E*-values, the reported *E*-value may not be consistent with the average number of false positives at all. For methods not reporting *E*-values, this does not show such methods can have the correct statistics either.

4. We are not sure what does the reviewer mean. *P*-value is the probability of obtaining a result at least as extreme as a given reference data point assuming that the data point was the result of chance alone. It is commonly used for statistical hypothesis testing indeed, but the meaning of the *P*-value is still a probability.

5. The background frequency is needed to *generate *a random protein/peptide database that we used for statistical calibration. It is not something that we employed for peptide scoring. Since the calibration is intended for general MS^2 ^search that may be used for studying different organisms, we choose the Robinson-Robinson frequency to avoid biasing towards the average amino acid frequencies of a certain organism.

### Reviewer's Report 2

Sent to the reviewer on August 29th, 2007. Review report received on September 28th, 2007. Review by Alexey Nesvizhskii (nominated by King Jordan), Department of Pathology, University of Michigan, Ann Arbor

The manuscripts deals with the problem of computing confidence measures for peptide assignments to tandem mass (MS/MS) spectra produced by database search programs such as SEQUEST, Mascot, OMSSA and etc. The authors describe a method for converting the MS/MS database search scores reported by each tool (expectation values, or raw scores such as cross-correlation score) into 'calibrated E-values'. The main purpose of this conversion is to reduce the dependence of the score on the size of the searched database and to allow more transparent comparison between different search algorithms. The calibration is achieved with a help of training datasets searched against databases of various sizes using each search tools that is being calibrated. While this work should be of interest to researchers directly involved in the development of database search tools and statistical data validation methods, several factors limit the significance of this work and its practical utility. More specifically:

1. The scope of this work is very limited in that it focuses only on the calibration of the primary MS/MS database search score. In practice, this score represent only one of the many factors that are used for accessing the validity of a peptide assignment. Other discriminative information includes the mass accuracy, presence of missed cleavage sites, number of peptide termini consistent with the enzymatic cleavage, and etc. Furthermore, secondary scores such as Delta score (difference between the top and the second best score) are often used as they add to discrimination. Thus, even if the calibration as proposed in the manuscript can be used to make the score more accurate, its power to discriminate between correct and incorrect identifications would still be low compared to other existing methods that utilize multiple discriminant parameters (see also point 2 below).

2. There is a large body of literature on methods for assigning confidence measures to peptide identifications, for a recent review see e.g. Nesvizhskii et al., Analysis and Statistical Validation of Proteomics Datasets Generated by Mass Spectrometry, Nature Methods 4, 787 – 797 (2007). Several methods for computing peptide probabilities and or False Discovery Rates (FDR) have been described and are commonly used. The authors should discuss how they work on single-spectrum measures (expectation values) is related to global methods such as the Empirical Bayes approach of PeptideProphet and target-decoy strategy for FDR estimation.

3. The practical utility of the proposed method (calibration performed by each individual lab) is questionable. Furthermore, there is enough variation between different datasets generated on the same type of mass spectrometer in the same lab due to differences in sample preparation protocols, different organisms that are studies, and the overall quality of the data.

4. The comparison between the theoretical and calibrated E-values (Figs. 1 and 2) should focus on the most relevant region of low error rates (E value of less than 0.1). In that region, performance of the calibrated scores appears to be not as good. Same applies to the true positive ratio plot (Fig. 3).

### Author's Response

1. The reviewer might have missed the key point of our protocol. To calibrate the statistics using scores is just a demonstration of what one may do to improve statistical accuracy. For any given search method, if feeding more information to a discriminant function can enhance the separation between true and false positives, one may simply calibrate/convert the output of the discriminant function to the standardized *E*-value. That is, developers of different search methods may use our protocol to their best advantages: getting the best performance for their method with accurate statistics.

2. We are well aware of the literatures attempting to address the issue of statistics in peptide identification. As for the particular reference mentioned by the reviewer, which was published online one day before the reviewer sent in his comments, we find that the questions we raised in this manuscript were also deemed important there as well. The reason we chose not to pursue global Bayes method and False Discovery Rate (FDR) type of analysis is simple. The noise in an MS^2 ^spectrum is spectrum-specific. Attempting to obtain a prior for *all *spectra in a given experiment is not ideal for capturing this virtue. As for the FDR type of analysis, we simply note the following. Although any hit on the decoy database is a false positive, hits in the target database are not necessarily true positives only. For example, for a given spectrum generated by a peptide contained in the target database, a search method may report significant hit on the target database but with a *different *peptide reported. This misidentification in the target database may vary from method to method, and thus hinder the use of FDR as a common ground to compare search results of various search methods.

3. In addition to issues mentioned by the reviewer, we note that there may exist more problems such as the generic variations within the same dataset arising from the difference in physical/chemical properties of the peptides. Furthermore, the data collection mode will also influence the calibration. For example, we have no reason to believe that the calibration done for profile mode data can be used directly to provide standardized statistics. However, in our view, this is a merit of our method. One should not anticipate to do the calibration once and then forget about it. When the experimental condition changes, a new calibration is called for. Below we address each issue raised by the reviewer specifically.

When analyzing data obtained under different sample preparation, a user is encouraged to recalibrate. Because each search method may be trained using dataset obtained under different conditions, there is no reason to believe that the *heuristic *scores reported can have consistency across samples prepared under very different conditions.

When different organisms are studied, if the sample preparation stays the same, it should not be necessary to do calibration again. In this case, peptides from all organisms will be in similar physical/chemical environments when the MS^2 ^experiments are performed. And any sensible search method will be able to provide consistent scores for peptide hits.

When experimental conditions and sample preparation protocols are fixed, the data quality variations may largely come from parent ion concentration as well as intrinsic spectrum-specific variation. In this case, there is no need to recalibrate. As for the former case, it was noted that the calibration was done with training datasets of various concentrations. As for the latter case, as noted by the reviewer, our calibrating the single-spectrum measures takes into account to some extent the spectrum-specific variation.

4. It is correct to focus on the low *E*-value regions if all one wants is to focus on the results of a single search method. If one were to look into the possibility of combining the search results from multiple search methods that are somehow orthogonal to each other, it becomes important to have the *E*-value correct even when it is close to 1. Another reason we can't go into much smaller *E*-value range is trivial. We have 10^4 ^spectra, thus *E*-values near or lower than 10^-4 ^are expected to follow the theoretical lines with much larger fluctuations. This is just the limit on the number of samples.

In the last panel of Figure 2, the *calibrated E*-value tracks well with the theoretical line in the lower *E*-value region. The reviewer might have misunderstood the other panels. Those other panels display either not-yet-calibrated *E*-values or the effective variable against the average number of false positives. The disagreement between the theoretical line and the data is what motivates us to *calibrate *the statistics. The same thing applies to Figure 3. The left panel of Figure 3 shows the results from uncalibrated *E*-values, while the right panel shows the results from the *calibrated E*-values. Although the right panel at low *E*-value region does not track well with theoretical line for many methods, we identify the problem to be the existence of protein clusters. Once implementing the cluster removal procedure, we show in Figure 4 that the agreement between theory and the data becomes much better.

### Reviewer's Report 3

Sent to the reviewer on September 5th, 2007. Review report received on October 8th, 2007. Review by Vineet Bafna, Department of Computer Science, UCSD.

Review for

"Calibrating E-values for MS2 library search", -Alves et al.

#### Synopsis

The paper addresses the issue of generating E-values for tools that search peptide databases to identify peptides, given MS2 spectra. The rationale for this is simple: the output of different search engines is currently not comparable, and cannot be combined easily. By generating a comparable E-value for each tool, a common cut-off can be specified.

#### Method

The authors make the reasonable assumption that the high scoring tail of the score distribution should ideally follow an exponential distribution. The authors construct a large random database which has been filtered to remove all true peptides. For each tool, they plot the cumulative number of hits (all false-positives) against a putative E-value. For tools that report E-values directly, it is used as is. For tools that report a score S, the putative E-value exp(-S) is used. If the E-value was a correct measure, the plot should be a straight line. If it is not, a suitable calibration is applied to make it linear. This calibration is the novel part of the paper.

#### Critique

1. The calibration is applied to 7 distinct tools with very different approaches to score computation. In each case, the authors show that with suitable recalibration, the computed E-value is reasonably predictive of the number of hits that would occur just by chance. This is tested using a number of plots. The recalibration transformations are usually straightforward, but can be complex in some cases.

Unfortunately, the authors do not comment on the effort needed for each new tool, and whether the calibration could be automated. It appears that the recalibration can only be done by experts, which limits the usefulness of the proposed method. Also, while the authors find good success on the tools that were attempted, it is not clear if other tools will have scores that can be scaled to fit an exponential distribution.

2. As a second and important criticism, the authors perhaps overstate the value of a statistical E-value test, citing earlier work of Fenyo and Beavis. To explain this point, a comparison must be made against sequence searching tools (such as BLAST) which also rely upon computation of E-values. The goal with sequence homology search is to find other 'similar' sequences. Indeed Every sequence that is statistically significantly similar to the query sequence is of potential interest, and should be reported.

The aim of MS2 searches is quite different. Here one aims to find the single peptide that has generated the spectrum. Consider for example a peptide 'ABCDE' that has a low E-value. If there is a second peptide ACBDE, it will have a very similar spectrum, and will show up as having a statistically significant E-value. Unfortunately, unlike sequence homology searches, this is bad news. The presence of two strong hits REDUCES the confidence in any one hit being the right one. This problem is also magnified by the scale of the searches. It is usual to search with thousands of query spectra, and only expect to have one correct hit for each. Thus, the output of a number of low E-value peptides for each spectra defeats the purpose of automated identification.

For this reason, many MS2 algorithms have a second, 'validation' step after the scoring. The goal of validation is not to identify a cut-off for statistical significance, but to compute a probability that the top-scoring peptide is the correct one. Thus for example, SEQUEST uses the delta-score, and Inspect uses a second probability generated by applying a statistical discrimination function against features like MQscore and others. Surprisingly the authors ignore these second level analyses and use Xcorr, and MQscore as the output of these two tools, respectively. In my opinion, this diminishes the real contribution of the paper.

Apart from these criticisms, the paper is well written, and researched. The authors are careful to use multiple analyses, tools, and data-sets to support their arguments, and the calibration functions provided in the paper can be directly adapted to improve E-value computations, and to combine the output of different tools.

As a minor comment, a) the Inspect version used is much older version than the one currently available. If possible, some of the analyses should be redone with the newer version prior to publication. b) The word library in the title and elsewhere should be replaced by database. Conventional terminology uses library to mean library of spectra, and database to refer to a collection of sequences.

### Author's Response

#### Method

We *did not *assume the high scoring tail of the score distribution to be an exponential. The purpose of introducing the effective variable *e*^-*S *^for score-reporting methods is to obtain a monotonic transformation of the score that has a smaller value when the score is higher, mimicking the behavior of *E*-value. This transformation helps to calibrate the *E*-values.

#### Critique

1. First, as we have mentioned earlier, we did not assume or require the score distribution to be exponential to perform our calibration protocol. Second, the protocol is actually very simple and anybody could learn to do it easily. To make it more transparent, we have expanded on the explanation of the procedure and simplified the notation in Table 2. As one may see now, it is actually rather straightforward to (re)calibrate the statistics whenever needed.

2. Although ideally each spectrum is generated by one peptide, this is not always be the case due to inevitable coelutions coming from the fact that HPLC procedure runs only for a finite amount time. Furthermore, finding multiple hits for a given spectrum should not be regarded as a bad news. One should retrieve *all *equally likely hits based on the information content in the spectrum. Purposeful exaggeration on the separation between the best hit and the second best hit does NOT increase our confidence of the best hit being correct. If the use of additional information can improve the separation between the true positive and the false positives, one should include those information in the scoring and the calibration should be done based on the output of this new, more distinguishing discriminant function. However, absent a clear instruction from each software package regarding how to form the most powerful discriminant function, what we demonstrated in the paper should be regarded as a proof of principle. Users/developers interested in obtaining more accurate statistics are encouraged to calibrate the *best *discriminant function they come up with to obtain both good performance and good statistical accuracy.

#### Minor comments

a) The InsPecT version we used was the newest when we started the project in 2006. There are two reasons that we don't intend to do another full-scale study for InsPecT in this paper. First, the focus of this paper is to demonstrate the possibility of *E*-value calibration, not to rank which search engine has best performance. We don't see a qualitative difference may arise from a version change except the possibility that a new calibration might be necessary. Second, each search method is updating their version number now and then. We don't intend to do the calibration for each new version coming out. Instead, we encourage the users/developers of the software to perform the simple calibration steps proposed.

## Appendix

In this appendix, we provide an argument which gives rise to

TP(E≤Ec)TP(E≤Ec)+FP(E≤Ec)=e−Ec.
 MathType@MTEF@5@5@+=feaafiart1ev1aaatCvAUfKttLearuWrP9MDH5MBPbIqV92AaeXatLxBI9gBaebbnrfifHhDYfgasaacPC6xNi=xI8qiVKYPFjYdHaVhbbf9v8qqaqFr0xc9vqFj0dXdbba91qpepeI8k8fiI+fsY=rqGqVepae9pg0db9vqaiVgFr0xfr=xfr=xc9adbaqaaeGacaGaaiaabeqaaeqabiWaaaGcbaqcfa4aaSaaaeaacqWGubavcqWGqbaucqGGOaakcqWGfbqrcqGHKjYOcqWGfbqrdaWgaaqaaiabdogaJbqabaGaeiykaKcabaGaemivaqLaemiuaaLaeiikaGIaemyrauKaeyizImQaemyrau0aaSbaaeaacqWGJbWyaeqaaiabcMcaPiabgUcaRiabdAeagjabdcfaqjabcIcaOiabdweafjabgsMiJkabdweafnaaBaaabaGaem4yamgabeaacqGGPaqkaaGaeyypa0Jaemyzau2aaWbaaeqabaGaeyOeI0Iaemyrau0aaSbaaeaacqWGJbWyaeqaaaaacqGGUaGlaaa@5059@

For a given spectrum and a score cutoff S_*c*_, one may ask what is the expected number of random hits (false positives) with score larger than or equal to S_*c*_. This is by definition the *E*-value associated with score cutoff S_*c*_. For a random protein database, each peptide is independent of the others. As a consequence, one may view the event of observing *k *random hits with score higher than S_*c *_as a Poisson process with expectation value *E*(S_*c*_). That is, the probability of obtaining *k *random peptides with *E*-values less than or equal to *E*_*c *_is given by

(Ec)kk!e−Ec.
 MathType@MTEF@5@5@+=feaafiart1ev1aaatCvAUfKttLearuWrP9MDH5MBPbIqV92AaeXatLxBI9gBaebbnrfifHhDYfgasaacPC6xNi=xI8qiVKYPFjYdHaVhbbf9v8qqaqFr0xc9vqFj0dXdbba91qpepeI8k8fiI+fsY=rqGqVepae9pg0db9vqaiVgFr0xfr=xfr=xc9adbaqaaeGacaGaaiaabeqaaeqabiWaaaGcbaqcfa4aaSaaaeaacqGGOaakcqWGfbqrdaWgaaqaaiabdogaJbqabaGaeiykaKYaaWbaaeqabaGaem4AaSgaaaqaaiabdUgaRjabcgcaHaaacqWGLbqzdaahaaqabeaacqGHsislcqWGfbqrdaWgaaqaaiabdogaJbqabaaaaiabc6caUaaa@3A67@

And the probability of observing one or more such random hits is given by

∑k=1∞(Ec)kk!e−Ec=1−e−Ec.
 MathType@MTEF@5@5@+=feaafiart1ev1aaatCvAUfKttLearuWrP9MDH5MBPbIqV92AaeXatLxBI9gBaebbnrfifHhDYfgasaacPC6xNi=xI8qiVKYPFjYdHaVhbbf9v8qqaqFr0xc9vqFj0dXdbba91qpepeI8k8fiI+fsY=rqGqVepae9pg0db9vqaiVgFr0xfr=xfr=xc9adbaqaaeGacaGaaiaabeqaaeqabiWaaaGcbaWaaabCaeaajuaGdaWcaaqaaiabcIcaOiabdweafnaaBaaabaGaem4yamgabeaacqGGPaqkdaahaaqabeaacqWGRbWAaaaabaGaem4AaSMaeiyiaecaaiabdwgaLnaaCaaabeqaaiabgkHiTiabdweafnaaBaaabaGaem4yamgabeaaaaaaleaacqWGRbWAcqGH9aqpcqaIXaqmaeaacqGHEisPa0GaeyyeIuoakiabg2da9iabigdaXiabgkHiTiabdwgaLnaaCaaaleqabaGaeyOeI0Iaemyrau0aaSbaaWqaaiabdogaJbqabaaaaOGaeiOla4caaa@4962@

Or equivalently, the probability of not getting any false positive hits with *E *≤ *E*_*c *_is given by e−Ec
 MathType@MTEF@5@5@+=feaafiart1ev1aaatCvAUfKttLearuWrP9MDH5MBPbIqV92AaeXatLxBI9gBaebbnrfifHhDYfgasaacPC6xNi=xI8qiVKYPFjYdHaVhbbf9v8qqaqFr0xc9vqFj0dXdbba91qpepeI8k8fiI+fsY=rqGqVepae9pg0db9vqaiVgFr0xfr=xfr=xc9adbaqaaeGacaGaaiaabeqaaeqabiWaaaGcbaGaemyzau2aaWbaaSqabeaacqGHsislcqWGfbqrdaWgaaadbaGaem4yamgabeaaaaaaaa@311D@. In the event when true peptides are all in the database, or equivalently each reported hit list contains at least a true positive, the quantity e−Ec
 MathType@MTEF@5@5@+=feaafiart1ev1aaatCvAUfKttLearuWrP9MDH5MBPbIqV92AaeXatLxBI9gBaebbnrfifHhDYfgasaacPC6xNi=xI8qiVKYPFjYdHaVhbbf9v8qqaqFr0xc9vqFj0dXdbba91qpepeI8k8fiI+fsY=rqGqVepae9pg0db9vqaiVgFr0xfr=xfr=xc9adbaqaaeGacaGaaiaabeqaaeqabiWaaaGcbaGaemyzau2aaWbaaSqabeaacqGHsislcqWGfbqrdaWgaaadbaGaem4yamgabeaaaaaaaa@311D@ becomes the probability of getting a true positive hit with *E*-value smaller than or equal to *E*_*c*_.

If we have a collection of *N *hits with *E*-values less than or equal to *E*_*c*_, then the total number of true positives is expected to be *N *e−Ec
 MathType@MTEF@5@5@+=feaafiart1ev1aaatCvAUfKttLearuWrP9MDH5MBPbIqV92AaeXatLxBI9gBaebbnrfifHhDYfgasaacPC6xNi=xI8qiVKYPFjYdHaVhbbf9v8qqaqFr0xc9vqFj0dXdbba91qpepeI8k8fiI+fsY=rqGqVepae9pg0db9vqaiVgFr0xfr=xfr=xc9adbaqaaeGacaGaaiaabeqaaeqabiWaaaGcbaGaemyzau2aaWbaaSqabeaacqGHsislcqWGfbqrdaWgaaadbaGaem4yamgabeaaaaaaaa@311D@. Note that the total number of true positives plus total number of false positives will be *N*, *i.e.*, *TP*(*E *≤ *E*_*c*_) + *FP*(*E *≤ *E*_*c*_) = *N *. Consequently, we obtain

TP(E≤Ec)TP(E≤Ec)+FP(E≤Ec)=e−Ec.
 MathType@MTEF@5@5@+=feaafiart1ev1aaatCvAUfKttLearuWrP9MDH5MBPbIqV92AaeXatLxBI9gBaebbnrfifHhDYfgasaacPC6xNi=xI8qiVKYPFjYdHaVhbbf9v8qqaqFr0xc9vqFj0dXdbba91qpepeI8k8fiI+fsY=rqGqVepae9pg0db9vqaiVgFr0xfr=xfr=xc9adbaqaaeGacaGaaiaabeqaaeqabiWaaaGcbaqcfa4aaSaaaeaacqWGubavcqWGqbaucqGGOaakcqWGfbqrcqGHKjYOcqWGfbqrdaWgaaqaaiabdogaJbqabaGaeiykaKcabaGaemivaqLaemiuaaLaeiikaGIaemyrauKaeyizImQaemyrau0aaSbaaeaacqWGJbWyaeqaaiabcMcaPiabgUcaRiabdAeagjabdcfaqjabcIcaOiabdweafjabgsMiJkabdweafnaaBaaabaGaem4yamgabeaacqGGPaqkaaGaeyypa0Jaemyzau2aaWbaaeqabaGaeyOeI0Iaemyrau0aaSbaaeaacqWGJbWyaeqaaaaacqGGUaGlaaa@5059@
